# Previous Exposure to Statin May Reduce the Risk of Subsequent Non-Hodgkin Lymphoma: A Nationwide Population-Based Case-Control Study

**DOI:** 10.1371/journal.pone.0139289

**Published:** 2015-10-01

**Authors:** Shih-Feng Cho, Yi-Hsin Yang, Yi-Chang Liu, Hui-Hua Hsiao, Chiung-Tang Huang, Cheng-Han Wu, Yu-Fen Tsai, Hui-Ching Wang, Ta-Chih Liu

**Affiliations:** 1 Graduate Institute of Clinical Medicine, College of Medicine, Kaohsiung Medical University, Kaohsiung, Taiwan; 2 Division of Hematology and Oncology, Department of Internal Medicine, Kaohsiung Medical University Hospital, Kaohsiung Medical University, Kaohsiung, Taiwan; 3 School of Pharmacy, Kaohsiung Medical University, Kaohsiung, Taiwan; University of British Columbia, CANADA

## Abstract

**Background:**

The purpose of this study was to investigate the association between previous exposure to statins and the risk of non-Hodgkin lymphoma (NHL).

**Methods:**

This nationwide population-based case–control study was conducted using the National Health Insurance Research Database of Taiwan. The NHL group consisted of the patients with a first-time diagnosis of NHL between 2005 and 2008. The cases of the control group were pair-matched to the NHL group according to sex, year of birth and date of NHL diagnosis (index date). The statin administration data from both groups were retrospectively collected from the index date to January 1, 1996. The cumulative defined daily dose (cDDD) was estimated to evaluate the statin exposure. Adjusted odds ratios (ORs) and 95% confidence intervals (CIs) were estimated using multivariate logistic regression.

**Results:**

The study population was composed of 1715 NHL patients and 16942 control subjects. The analysis revealed that previous statin administration was associated with a reduced risk of subsequent NHL with an adjusted OR of 0.52 (95% CI, 0.43–0.62). Additionally, there was a dose-response relationship between statin administration and the risk of NHL. The adjusted ORs were 0.63 (95% CI, 0.46–0.86), 0.58 (95% CI, 0.42–0.79), 0.51 (95% CI, 0.38–0.67), and 0.36 (95% CI, 0.24–0.53) for the subjects with statin administrations of fewer than 28, 28 to 90, 91 to 365, and more than 365 cDDDs, respectively, relative to the subjects without any statin administration.

**Conclusions:**

The results of this study suggest that previous statin administration is associated with a lower risk of subsequent NHL. As statins are widely used medications, the magnitude of the risk reduction may have a substantial influence on public health. Further studies to confirm our findings are warranted.

## Introduction

Inhibitors of 3-hydroxy-3-methylglutary–coenzyme A reductase (HMG-CoA) are also known as statins and are common cholesterol-lowering medications that are widely used for primary and secondary prevention of cardiovascular disease and stroke[1–3].

In addition to statins’ cholesterol-lowering effect, several in-vitro and in-vivo studies have suggested that statins may have anticancer properties that are mediated via various mechanisms, such as the arrest of the cell cycle[4,5], induction of apoptosis[6–8], and inhibition of tumor growth metastasis [9–11]. Several meta-analyses and observational studies have shown that statin administration is either not associated with an increase in the development of cancer[12–15] or even associated with a decrease in risk of the development of cancer[16–20]. Regarding the administration of statins and the risk of hematologic malignancy, one meta-analysis suggested that statins may have chemopreventive effects and reported a risk reduction of 19%[21]. However, another meta-analysis did not support this result[22].

Non-Hodgkin lymphoma (NHL) is the most common hematologic malignancy in the adult population, and its incidence has increased significantly worldwide[23–25]. With respect to its pathophysiology, the development of NHL has been associated with chronic inflammatory statuses, such as chronic bacterial or viral infections, and autoimmune diseases. The association between the administration of statins and the risk of NHL was evaluated in a case-control study conducted in European countries that revealed that statin administration reduced the risk of NHL by approximately 40%[26]. However, previous epidemiologic studies have revealed that the distribution of malignant lymphomas in Asian countries differs from that in Western countries. These findings indicate that the etiologies of malignant lymphomas may exhibit racial and geographic differences[27–30]. In terms of the association between statin administration and the risk of hematologic malignancies, particularly NHL, the majority of studies have been performed in Western countries, and related data for Asian countries are relatively rare.

As numerous people use statins on a long-term basis in Taiwan, we conducted this nationwide population-based case-control study to evaluate the association between previous statin usage and the subsequent development of NHL.

## Participants and Methods

### Data Source

The primary data source for this study was the National Health Insurance Research Database (NHIRD) of the National Health Insurance (NHI) of Taiwan. The NHI program is a government-run, single-payer mandatory health insurance program that was initiated in 1995 and covers of all forms of medical services including inpatient and outpatient care, dental care, preventive medicine, childbirth, Chinese medicine, home care, physical therapy and rehabilitation for chronic mental illnesses. The NHIRD was built by National Health Research Institute (NHRI) of Taiwan in 1997. The content of the NHIRD includes the original claim data from the NHI and the demographic information of all NHI beneficiaries. Because more than 98% of Taiwan’s population participates in the NHI program, the NHIRD was able to provide the most comprehensive information for this study[31]. Several previous high-quality epidemiologic studies have been conducted using the NHIRD [32,33].

### Identification of the NHL Group

In this study, all NHL cases are collected from the database of Registry for Catastrophic Illness Patients, a subpart of the NHIRD. The Registry for Catastrophic Illness Patients is a nationwide registration system established by the NHIRD for the patients who suffered from certain severe illnesses, such as autoimmune diseases, end-stage renal disease and malignant diseases. The patients with above major diseases are able to apply for catastrophic illness registration to waive all co-payments of the NHI program when seeking medical services for the catastrophic illness. The approval for a certificate of catastrophic illness involves a strict review process conducted by the Department of Health of Taiwan. For patients with NHL, the certificates of catastrophic illness were issued once the lymphoma was proved by pathology. The database of Registry for Catastrophic Illness Patients includes information, such as diagnostic codes in the International Classification of Disease, 9th Revision, Clinical Modification (ICD-9-CM) format, the date of the diagnosis of catastrophic illness, the date of death, dates of each clinic visit, data of prescriptions, amounts of expenditure, and inpatient/outpatient claims, for all the beneficiaries with catastrophic illnesses from 1996 to 2008.

The patients with NHL were identified using the ICD-9-CM codes of the NHL (i.e., 200.0–200.8, 202.0–202.9). The NHL group was composed of patients aged 20 years and older who were first diagnosed with NHL between the 1^st^ of January 2005 and the 31^st^ of December 2008. The index dates of the NHL group were taken as the dates on which NHL was diagnosed. The administrative data for the NHL group were composed of all of the original outpatient and inpatient care data since 1996. We excluded patients who had antecedent or co-existing malignant diseases (140.0–199.1) or HIV infection (042) that were diagnosed before the index date.

### Identification of the Control Group

The data for the control group were retrieved from the Longitudinal Health Insurance Database 2005 (LHID2005), which is a subpart database of NHIRD that contains randomly selected representative data from 1,000,000 patients among all NHI enrollees from the year 2005 registry that were selected by a systematic sampling method for research purposes. According to the report of the NHIRD, there were no significant differences in the demographic information of the LHID2005 sample and the entire NHI database. This database is composed of all of the original medical claims related to the 1,000,000 enrollees’ historical outpatient and inpatient care under the NHI program from 1996 to 2008.

The control group was composed of patients who were hospitalized with diagnoses unrelated to statin administration, including orthopedic conditions, trauma (excluding wrist and hip fractures), and other conditions (acute infection, hernia, kidney stones, and cholecystitis). We excluded patients with wrist and hip fractures that were diagnosed before the index date because previous articles have shown that statin users are at a reduced risk of osteoporosis [34–36]. The patients with a previous cancer diagnosis or HIV infection were also excluded.

The patients in the control group were randomly pair-matched to the patients with NHL according to sex, year of birth and index date (10 controls for every NHL case). The index dates (i.e., the date of hospital admission) of the control subjects were within the same month of the index dates of their matched NHL subjects.

### Evaluation of Previous Statin Exposure

To evaluate the association between the statin dosage and NHL, the data, including the date the statin was prescribed, the daily dose, and the number of days administered, were collected. All subjects in both groups were traced back to evaluate the previous statin usages from the index date to January 1, 1996. We utilized the defined daily dose (DDD), which is recommended by the World Health Organization as a unit for measuring the prescribed amount of a drug. The DDD was calculated according to the following formula: (total amount of drug)/(amount of drug in a DDD) = number of DDDs. To evaluate the durations of exposures, the cumulative DDDs (cDDDs) were calculated to measure the combinations of doses and days of the statins (specifically, lovastatin, pravastatin, rosuvastatin, fluvastatin, simvastatin, and atorvastatin) from the 1^st^ of January 1996 to the index date.

### Identification of Potential Confounders

We identified the documented risk factors for NHL as the potential confounders that were recorded between the index date and the 1^st^ of January 1996, which included autoimmune disease (rheumatoid arthritis (714), systemic lupus erythematosus (710.0) and sicca syndrome (710.2))[37], hepatitis B infection (070.2, 070.3, V02.61) [38], hepatitis C infection (070.7, 070.41, 070.44, 070.51, 070.54, V02.62) [39,40] and diabetes mellitus (250) [41,42]. Some confounders associated with statin administration, such as hypertension (201–204) and hyperlipidemia (272), were also added for adjustment.

In terms of environmental factors, we adjusted for the urbanization level. According to the standards established by the NHRI, all 365 towns in Taiwan are stratified into 5 levels with level 1 representing the most urbanized areas. The criteria for determining the urbanization level included the density of population (persons/km^2^), the number of physicians (per 100,000 people), the percentage of workers in the agricultural field, the percentage of residents with a college education, and the percentage of the elderly people (over 65 years of age).

We also identified the following medications that could have potentially confounded the association between the use of statins and the risk of cancer: nonsteroidal anti-inflammatory drugs (NSAIDs), cyclooxygenase-2 (COX-2) inhibitors, aspirin, angiotensin-converting enzyme inhibitors (ACEI), and other lipid-lowering drugs (including fibrate, niacin, bile acid-binding resins, and others). The subjects who had taken the above-mentioned medications for at least one prescription during the 1-year period prior to the index date were defined as users.

### Statistical Analysis

The chi-square test (χ2 test) was performed to compare the frequencies of each of the categorical variables. To evaluate the association between the statin and NHL, the study population was categorized as non-users (subjects without prescriptions for any statin at any time between the index date and the 1^st^ of January 1996) and statin users (subjects with at least one prescription for statin during the same period). The statin users were further categorized into four groups based on the cDDDs (<28, 28 to 90, 91 to 365, and > 365 cDDDs). A multivariate logistic regression was performed to estimate the relative magnitude in relation to statin administration. The odds ratios (ORs) and their 95% confidence intervals (95% CIs) were calculated using statin non-users as the reference. The NHL risk analyses were adjusted for gender, age, urbanization level, diabetes mellitus, HBV infection, HCV infection, hypertension, hyperlipidemia, autoimmune diseases (RA, SLE and sicca syndrome), and the use of ACEI, NSAID, COX-2 inhibitors and aspirin, fibrate and other lipid-lowering drugs. The analyses were performed using the SAS statistical package (version 9.3, SAS Institute, Cary, NC). All of the statistical tests were two-sided. Values of *P* < 0.05 were considered statistically significant.

### Ethical Approval

The NHIRD is a completely de-identified and encrypted database for use in research only. This study was conducted in accordance with the Declaration of Helsinki. The study was also reviewed and approved by the Institutional Review Board of Kaohsiung Medical University Hospital (KMUH-IRB-EXEMPT-20140027).

## Results

**Fig 1:**
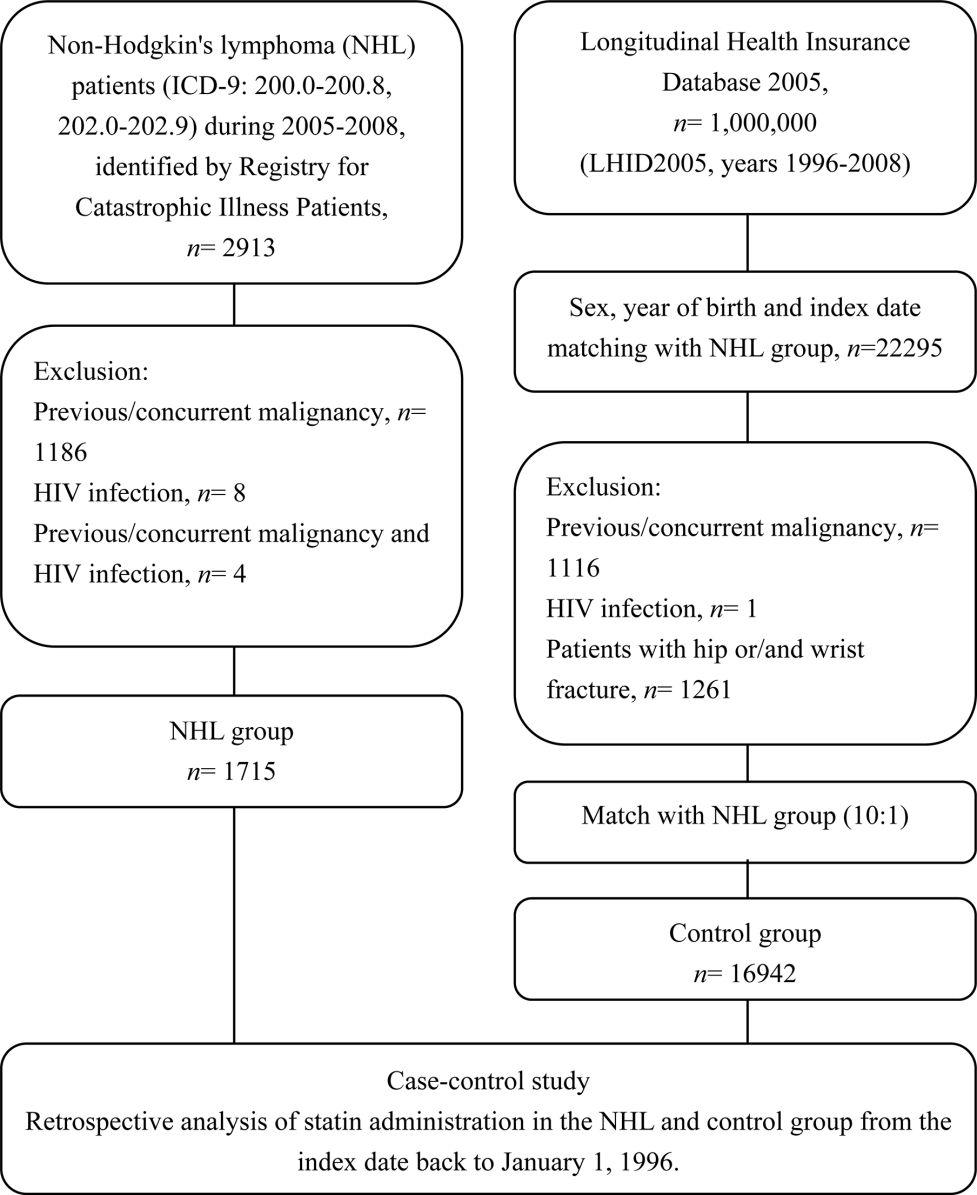
Flow chart summarizing the data acquisition for the NHL and control groups.

Between 2005 and 2008, 2913 patients were diagnosed with NHL. After excluding the patients with previous malignancies and HIV infection, a total of 1715 patients with NHL were enrolled in this study. After matching for sex, year of birth and index date, we randomly selected 16,942 control subjects, approximated 91.95% as 10:1 matched with the NHL patients. The research design of this study is illustrated in Fig 1.

The demographic data for each of the groups are listed in Table 1. In terms of the medical histories and risk factors for lymphoma, significantly higher rates of HBV infection, HCV infection, diabetes mellitus, hypertension, hyperlipidemia, rheumatoid arthritis, systemic lupus erythematosus and sicca syndrome were present in the NHL group. Regarding the selected medical conditions, the NHL group exhibited a significantly lower rate of prior statin administration and higher rates of NSAID and COX-2 inhibitors administration. There were no significant differences in the usages of ACEI, aspirin or other lipid-lowering agents between these two groups (Table 2).

**Table 1:** Demographic characteristics of the NHL group and the matched control group.

Variables	Total number (*n* = 18657)	NHL group (*n* = 1715)	Control group (*n* = 16942)	*P*-value
Age (mean ± SD)	58.76±16.53	58.99±16.62	58.73±16.52	1.000
20–49 years of age (*n*, %)	5421(29.06)	493(28.75)	4928 (29.09)	
50–59 years of age (*n*, %)	4010(21.49)	365 (21.28)	3645 (21.51)	
60–69 years of age (*n*, %)	3556(19.06)	325 (18.95)	3231 (19.07)	
70–79 years of age (*n*, %)	3755(20.13)	347(20.23)	3408 (20.12)	
≥ 80 years of age (*n*, %)	1915(10.26)	185 (10.79)	1730 (10.21)	
Male sex (*n*, %)	10640 (57.03)	979 (57.08)	9661(57.02)	1.000
Urbanization level^*^				<0.001
1 (*n*, %)	4680 (25.08)	412 (24.02)	4268 (25.19)	
2 (*n*, %)	1439 (7.71)	94 (5.48)	1345 (7.94)	
3 (*n*, %)	773 (4.14)	64 (3.73)	709 (4.18)	
4 (*n*, %)	8041 (43.10)	672 (39.18)	7369 (43.49)	
5 (*n*, %)	3652(19.57)	469 (27.35)	3183 (18.79)	
Medical diseases				
HBV (*n*, %)	188 (1.00)	59 (3.44)	129 (0.76)	<0.001
HCV (*n*, %)	85 (0.46)	22 (1.28)	63 (0.37)	<0.001
DM (*n*, %)	1510 (8.09)	256 (14.93)	1254 (7.40)	<0.001
Hypertension (*n*, %)	3541 (18.98)	524 (30.55)	3017 (17.81)	<0.001
Hyperlipidemia (*n*, %)	1474 (7.90)	202 (11.78)	1272 (7.51)	<0.001
RA (*n*, %)	134 (0.72)	37 (2.16)	97 (0.57)	<0.001
SLE (*n*, %)	17 (0.09)	10 (0.58)	7 (0.04)	<0.001
Sicca syndrome (*n*, %)	65 (0.35)	15 (0.87)	50 (0.29)	<0.001

*72 subjects, including 4 in the NHL group and 68 in the control group, were not stratified. HBV, hepatitis B virus; HCV, hepatitis C virus; DM, diabetes mellitus; RA, rheumatoid arthritis; SLE, systemic lupus erythematosus

**Table 2:** Histories of drug exposure of the NHL matched control groups.

Variables	Total number (*n* = 18657)	NHL group (*n* = 1715)	Control group (*n* = 16942)	*P*-value
Any statin use (*n*, %)	2760 (14.79)	200 (11.66)	2560 (15.11)	<0.001
Statin				
Lovastatin (*n*, %)	1090 (5.84)	80 (4.66)	1010 (5.96)	0.029
Pravastatin (*n*, %)	526 (2.82)	34 (1.98)	492 (2.90)	0.028
Rosuvastatin (*n*, %)	334 (1.79)	17 (0.99)	317 (1.87)	0.009
Fluvastatin (*n*, %)	568 (3.04)	34 (1.98)	534 (3.15)	0.007
Simvastatin (*n*, %)	935 (5.01)	62 (3.62)	873 (5.14)	0.005
Atorvastatin (*n*, %)	1205 (6.46)	90 (5.25)	1115 (6.58)	0.032
Other Drugs				
Aspirin (*n*, %)	2607 (13.97)	232 (13.53)	2375(14.02)	0.577
NSAID (*n*, %)	11069(59.33)	1215 (70.85)	9854(58.16)	<0.001
ACEI (*n*, %)	1630 (8.74)	144 (8.40)	1486 (8.77)	0.601
COX-2 inhibitor (*n*, %)	2181 (11.69)	248 (14.46)	1933 (11.41)	<0.001
Fibrate and other lipid-lower agents (*n*, %)	573 (3.07)	45 (2.62)	528 (3.12)	0.259

NSAID, non-steroidal anti-inflammatory drug; ACEI, angiotensin-converting enzyme inhibitor; COX-2, cyclooxygenase-2

The association between previous statin exposure and the risk of subsequent NHL was analyzed (Table 3). The numbers of subjects with any previous statin usage in the NHL and control groups were 200 (11.66%) and 2560 (15.11%), respectively. The analysis revealed that previous exposure to any statin was associated with a reduced risk of NHL with a crude OR of 0.74 (95% CI, 0.64–0.87). Additionally, there was a trend toward a decreased in the crude ORs for the NHL risk following the stratification of the users by the cumulative statin doses. After adjusting for the possible confounders (i.e., age, gender, urbanization level, diabetes mellitus, hypertension, hyperlipidemia, HBV infection, HCV infection, RA, SLE, sicca syndrome and the use of other lipid-lowering agents), the adjusted ORs remained significantly decreased (adjusted OR: 0.52; 95% CI, 0.43–0.62). When the statin users were further categorized by their cumulative doses, the inverse association between the administration of statins and the risk of NHL was found to be more significant at the higher cumulative doses of statins. The adjusted ORs were 0.63 (95% CI, 0.46–0.86), 0.58 (95% CI, 0.42–0.79), 0.51 (95% CI, 0.38–0.67), and 0.36 (95% CI, 0.24–0.53) for the cDDDs of statins of fewer than 28, 28 to 90, 91 to 365, and more than 365, respectively.

**Table 3:** Crude and adjusted odds-ratios of non-Hodgkin lymphoma (NHL) associated with previous statin administration during the follow-up period in the study cohort.

			Univariate			Multivariate		
	NHL group (*n* = 1715)	Control group (*n* = 16942)	Crude OR	95% CI	*P*-value	Adjusted* OR	95% CI	*P*-value
Overall								
Any statin use (vs. non-user)	200	2560	0.74	0.64–0.87	<0.001	0.52	0.43–0.62	<0.001
Cumulative use (vs. non-user)								
User (< 28 cDDDs)	48	560	0.81	0.60–1.10	0.177	0.63	0.46–0.86	0.004
28–90 cDDDs	51	599	0.81	0.60–1.08	0.151	0.58	0.42–0.79	<0.001
91–365 cDDDs	68	845	0.76	0.59–0.98	0.036	0.51	0.38–0.67	<0.001
>365 cDDDs	33	556	0.56	0.40–0.80	0.002	0.36	0.24–0.53	<0.001

OR, odds ratio; CI, confidence interval; cDDD, cumulative defined daily dose.

*Adjusted for age, gender, urbanization level, HBV infection, HCV infection, autoimmune diseases (RA, SLE and sicca syndrome), diabetes mellitus, hypertension, hyperlipidemia, and the uses of ACEI, NSAID, COX-2 inhibitors and aspirin, fibrate and other lipid-lowering drugs.

## Discussion

In this population-based case-control study, we retrospectively analyzed the histories of statin administration in the NHL and control groups. The results indicated that previous statin administration was associated with a 48% reduction in the NHL risk compared with the patients who did not take any statins after controlling for the potential confounders. Additionally, this study also revealed a trend toward a greater NHL risk reduction with longer-term and more frequent prescriptions of statins. An early population-based case-control study conducted by Fortuny J *et al*. in Europe reported a risk reduction magnitude of 40%, which agrees with our results[26]. Unlike our study, that study did not find a greater reduction in the risk of lymphoma in the patients with prolonged statin administration durations. This difference may potentially have been caused by the different confounding variables that were adjusted for in these two studies. Moreover, the number of cases and the percentage of statin users were higher in our study possibly because statins may have been more frequently prescribed in recent years.

The details of the mechanisms by which statin use may decrease the risk of NHL are not well understood, but some potential mechanisms may support the results of the present study. First, statins have been shown to exhibit anti-inflammatory properties [43,44]. Previous research has shown that chronic inflammation can lead to downstream oncogenic mutations in signaling pathways that have been linked to the development of various tumors, including lymphomas[45–47]. Second, statins have various antitumor properties related to many cancers. A previous animal model study revealed that atorvastatin can inactivate the RAS and ERK1/2 signaling pathways and inhibit the expression of MYC oncogene, which are thought to be closely associated with the development of malignant lymphomas[48]. Another study indicated that statins exert anti-lymphoma effects via the induction of apoptosis, which is associated with an increased generation of reactive oxygen species, activation of p38 and suppression of the AKT and ERK pathways[49]. Additionally, previous studies have revealed that patients who adhere to chronic preventive medicine are more likely to seek healthier lifestyles and preventive medical services, which may also partly explain the lower risk of NHL in long-term statin users[50,51].

This nationwide population-based case-control study has some strengths. First, our study used a computerized database that is population-based and highly representative. Additionally, because approximately 98% of the residents of Taiwan are of Han Chinese ethnicity, the potential confounds due to heterogeneous genetic backgrounds may have been reduced. Moreover, the diagnoses of NHL are believed to be correct due to the validation by the Registry for Catastrophic Illness Patients. Second, the present study demonstrated the dose-dependent nature of the effects of statins in the chemoprevention of NHL by analyzing the cumulative DDDs. Furthermore, we selected the control group from only the patients who had been hospitalized to ensure that the index dates were the same between the NHL and control groups. Because each of the groups in this study had similar durations of investigation, the potential association bias was minimized. Third, statins are available only by prescription in Taiwan. The data regarding statin administrations were collected from a historical database; therefore, the prescription data from before the dates of NHL diagnoses were all available, and thus recall bias was avoided.

This study also has some limitations. First, although we adjusted for the potential confounders in the statistical analysis, a number of possible unmeasured confounding variables, such as Epstein-Barr virus and *Helicobacter pylori* infection statuses, which are associated with NHL, were not available in this database. Second, we excluded the patients with HIV infection because of the very small number of cases. According to data from the Centers for Disease Control of Taiwan, the rate of new annual cases of HIV remained relatively low until the end of 2005. However, a previous case-control study revealed that statin administration may reduce the risk of NHL in HIV-positive patients [52]. Third, because we were unable to contact the patients directly about their use of statins due to the anonymization of their identification numbers, it is possible that we overestimated the administration of statins because we presumed that all of the prescribed statins were actually taken by the patients. Finally, we analyzed the data only in terms of the statins that were administered after 1996, and information about the prescription of statins prior to 1996 was not available. This lack of information may have resulted in the underestimation of the cDDDs and dose-response effects.

In summary, the results of our study revealed that previous statin administration was associated with a significant 48% reduction in the development of subsequent NHL. Additionally, the chemopreventive effects of statins increased with prolonged usage durations and increased dosages. Because statins are commonly prescribed and widely used medications, the magnitude of the risk reduction may have a substantial affect on public health. Additional studies, particularly prospective randomized trials, are warranted to confirm our findings.
